# Comparison of Interferon-γ Release Assay to Two Cut-Off Points of Tuberculin Skin Test to Detect Latent *Mycobacterium tuberculosis* Infection in Primary Health Care Workers

**DOI:** 10.1371/journal.pone.0102773

**Published:** 2014-08-19

**Authors:** Fernanda Mattos de Souza, Thiago Nascimento do Prado, Jair dos Santos Pinheiro, Renata Lyrio Peres, Thamy Carvalho Lacerda, Rafaela Borge Loureiro, Jose Américo Carvalho, Geisa Fregona, Elias Santos Dias, Lorrayne Beliqui Cosme, Rodrigo Ribeiro Rodrigues, Lee Wood Riley, Ethel Leonor Noia Maciel

**Affiliations:** 1 Laboratório de Epidemiologia of Universidade Federal do Espírito Santo, Vitória, Espírito Santo, Brazil; 2 Núcleo de Doenças Infecciosas of Universidade Federal do Espírito Santo, Vitória, Espírito Santo, Brazil; 3 Programa de Pós-graduação em Saúde Coletiva of Universidade Federal do Espírito Santo, Vitória, Espírito Santo, Brazil; 4 Programa de Controle de Tuberculose - Hospital Universitário Cassiano Antônio Moraes of Universidade Federal do Espírito Santo, Vitória, Espírito Santo, Brazil; 5 Division of Infectious Disease and Vaccinology, School of Public Health, University of California, Berkeley, California, United States of America; 6 Coordenador do Núcleo de Controle da Tuberculose - Secretaria Municipal de Saúde - Manaus, Amazonas, Brazil; 7 Departamento de Epidemiologia do Instituto de Medicina Social, Universidade Estadual do Rio de Janeiro, Rio de Janeiro, Brazil; 8 Estudantes de Graduação em Enfermagem e Obstetrícia da Universidade Federal do Espírito Santo, Vitória, Espírito Santo, Brazil; Public Health England, United Kingdom

## Abstract

**Background:**

An interferon-γ release assay, QuantiFERON-TB (QFT) test, has been introduced an alternative test for the diagnosis of latent *Mycobacterium tuberculosis* infection (LTBI). Here, we compared the performance of QFT with tuberculin skin test (TST) measured at two different cut-off points among primary health care work (HCW) in Brazil.

**Methods:**

A cross-sectional study was carried out among HCWs in four Brazilian cities with a known history of high incidence of TB. Results of the QFT were compared to TST results based on both ≥5 mm and ≥10 mm as cut-off points.

**Results:**

We enrolled 632 HCWs. When the cut-off value of ≥10 mm was used, agreement between QFT and TST was 69% (k = 0.31), and when the cut-off of ≥5 mm was chosen, the agreement was 57% (k = 0.22). We investigated possible factors of discordance of TST vs QFT. Compared to the TST−/QFT− group, risk factors for discordance in the TST+/QFT− group with TST cut-off of ≥5 mm included age between 41–45 years [OR = 2.70; CI 95%: 1.32–5.51] and 46–64 years [OR = 2.04; CI 95%: 1.05–3.93], BCG scar [OR = 2.72; CI 95%: 1.40–5.25], and having worked only in primary health care [OR = 2.30; CI 95%: 1.09–4.86]. On the other hand, for the cut-off of ≥10 mm, BCG scar [OR = 2.26; CI 95%: 1.03–4.91], being a household contact of a TB patient [OR = 1.72; CI 95%: 1.01–2.92] and having had a previous TST [OR = 1.66; CI 95%: 1.05–2.62], were significantly associated with the TST+/QFT− group. No statistically significant associations were found among the TST−/QFT+ discordant group with either TST cut-off value.

**Conclusions:**

Although we identified BCG vaccination to contribute to the discordance at both TST cut-off measures, the current Brazilian recommendation for the initiation of LTBI treatment, based on information gathered from medical history, TST, chest radiograph and physical examination, should not be changed.

## Introduction

Although the incidence of tuberculosis (TB) has gradually declined over the last 20 years worldwide, it remains a major infectious cause of morbidity and mortality in developing countries [1]. Health care workers (HCW) are one of the groups at risk of *Mycobacterium tuberculosis* (Mtb) infection, or latent TB infection (LTBI), due to their occupational exposure [2]–[4]. This risk has been associated with duration of exposure during their health care service, working in higher risk settings such as emergency rooms, inpatient units and laboratories, as well as delay in diagnosis and absence of work-related environmental preventive control measures [5]–[8].

Therefore, the screening of HCW for LTBI is critical in an infection control program [9]. Since 2004, the National Control Tuberculosis Program of Brazil redirected efforts for TB control from the inpatient setting to primary care clinics. With this change, the strategy emphasizes efforts on expanding case detection, improving treatment adherence and reducing treatment default [10]. In Brazil, biosafety guidelines are in place for hospital settings, but they are absent in other health care settings. Data from a previous study of tuberculin skin test (TST) survey carried out among HCW at primary care facilities in Brazil demonstrated a prevalence of LTBI of 26% [11]. Several limitations to the estimate of LTBI based on TST have been identified, which include cross-reaction from BCG and exposure to environmental mycobacteria in places like Brazil [12], .

Interferon-gamma release assays (IGRAs), based on the release of interferon-gamma (IFN-γ) by lymphocytes in response to specific Mtb antigens, were developed to overcome some of the above limitations of TST. One commercial IGRA, QuantiFERON test (QFT) is based on Mtb-specific antigens ESAT-6, CFP-10 and TB7-7, and is considered more specific than TST as the antigens used are not shared by any of the BCG vaccines or by most environmental mycobacteria [14].

According to the new Brazilian guidelines a TST cut-off point ≥5 mm should be considered as a positive result [15]. This change could potentially affect the agreement between TST and QFT results, especially in a TB-endemic setting where BCG is used. Here, we compared the performance of QFT to TST measured at two different cut-off points among primary HCWs, and assessed their concordance and discordance, as well as factors associated with these test results.

## Methods

### Study design and setting

A cross-sectional study was conducted from 2011 to 2012 in four Brazilian cities with a high incidence of TB: Vitória-ES (39.98/100,000), Cuiabá-MT (51.77/100,000), Salvador-BA (59.87/100,000) Manaus-AM (71.26/100,000) [16].

### Study population

The study population comprised primary HCW (physicians, nurses, nurse technicians and community health workers [CHW]). The exclusion criteria included known HIV status, HIV infection based on rapid test, prior TB, and being pregnant.

### Variables

The HCW interviews and demographic data, including factors associated with positive TST or QFT results were acquired in person by trained registered nurses (RN) (Questionnaire S1 and S2). These included gender, age (19–30; 31–35; 36–40; 41–45; 46–64 years), presence of BCG scar, professional category (physicians, nurse, nurse technician or CHW), work only at a primary health care, contact with a household member with TB, alcohol abuse, prior TST, smoker or ex-smoker, years served in health care profession at primary health care (<5 or ≥5 years) and comorbidity.

### Interferon-γ release assay

After the questionnaire was completed and a signed consent form obtained, 3 mL of blood was collected for the QuantiFERON TB Gold in-tube test (QFT) (1 mL in each tube). The test was performed according to the manufacturer's instructions (Cellestis Ltd, Carnegie, Victoria, Australia). The samples were transported to the reference laboratory at each capital (Municipal Laboratory Cuiaba-MT; Municipal Laboratory of Salvador-BA; Laboratory of Immunology of the Infectious Diseases Center of the Federal University of Espírito Santo, Vitória-ES and Manaus in the Laboratory of Microbiology of the Tropical Medicine Foundation Dr. Heitor Vieira Golden-Amazonas) within 4–6 h of collection and incubated for 16–24 h at 37°C. The samples were centrifuged at 3000× rcf (relative centrifugal force) for 15 min, and the collected plasma was stored at −20°C until use.

The samples collected in Cuiaba, Manaus and Salvador were transported in cryobox inside cooler containing ice packs to the Laboratory of Immunology, Infectious Diseases Center of Federal University of Espírito Santo in less than 6 hours, and stored at −20/−70°C until the IFN-γ assay was performed. The optical density (OD) of each test was read with a 450 nm filter with a 620 nm reference filter, with an ELISA plate reader.

Results were interpreted according to the manufacturer instructions. The cut-off value for a positive test was 0.35 IU/mL of IFN-γ in the plasma after stimulation, regardless of the result of the mitogen control. The result of the test was considered indeterminate if an antigen-stimulated sample tested negative and if the value of the positive control was less than 0.5 IU/ml after subtraction of the value of the nil control. Values in between were considered indeterminate.

The HIV rapid test was performed in the laboratory with this same blood sample (Rapid Check HIV 1 & 2/NDI-UFES Vitória–ES-Brazil).

### Tuberculin skin test

Immediately after the standardized interview was completed and blood was drawn for QFT, a Mantoux skin test containing 0.1 ml of PPD RT23 (Tuberculin PPD Evans 2 TU) was administered intradermally by a trained RN. The induration was measured after 48–72 hours after administration of the PPD and interpreted by the same nurses, according to Brazilian National Institute recommendation [15].

### Data Analysis

Results of each test were interpreted independently and the QFT results were interpreted without knowledge of the results of the TST. The concordance between the TST and QFT test results was measured by kappa (κ) statistics. A κ value of ≤0.4 was regarded as poor, >0.75 as excellent, and in between as fair to good agreement [17]. Furthermore, we applied the McNemar's test for evaluating the discordance. Factors associated with positive TST, QFT, TST+/QFT− and TST−/QFT+ results were evaluated by prevalence odds ratios (OR). A logistic regression model was used to adjust for exposure variables chosen on the basis of biological plausibility and on statistical and epidemiological criteria. A *p* value of less than 0.05 was defined to indicate a statistically significant difference in bivariate and multivariate analysis. Fisher's exact test was used to compare the discordant results (TST+/QFT− and TST−/QFT+) depending on the categorized IFN concentration.

### Ethical approval

The Universidade Federal do Espírito Santo (UFES) Institutional Review Board approved the study design under registration number 007/10. A written informed consent was obtained from all participating patients.

## Results

**Figure 1:**
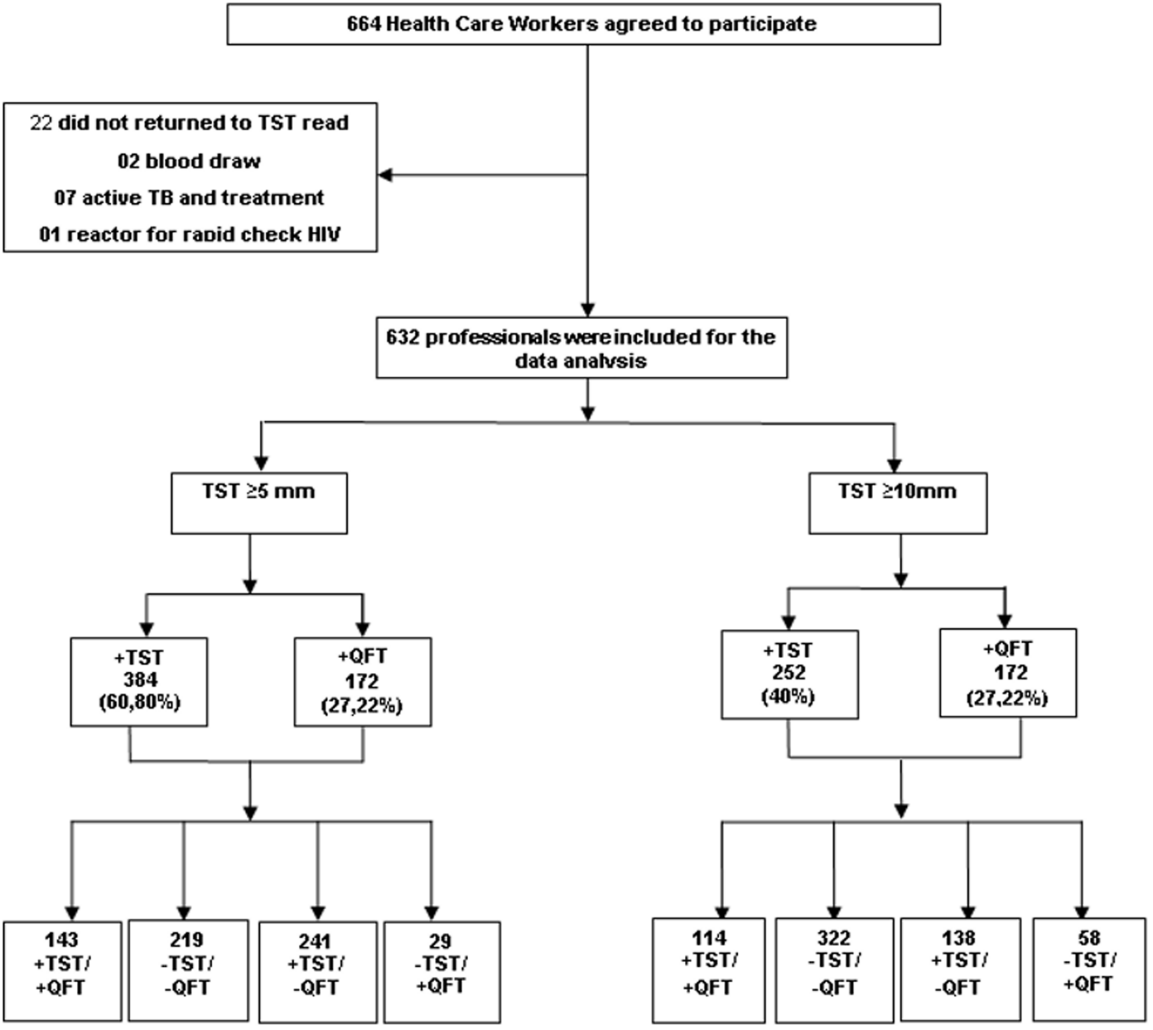
Study flow diagram. Tuberculin Skin Test-TST; Quantiferon TB Gold in tube test-QFT.

Between 2011 and 2012, 664 HCWs were enrolled (Figure 1); however, 22 (3.3%) participants were excluded because they did not return for TST reading, 2 (0.3%) refused to have blood drawn, 7 (1.1%) had active TB or were under TB treatment and 1 (0.2%) was HIV positive (positive rapid test).

Therefore, 632 (95%) of 664 HCW were included in this study. Median age was 42 years (40.8–42.4 years); 546 (86.4%) had vaccine BCG scars; and the median time of work as primary HCW was 9 years (range 1–39), 278 (46%) worked only at primary health care facilities. Of 354 (54%) HCW who worked in other health care facilities, 336 (90%) worked in a hospital ward. Of these, 67 (20%) worked in a pulmonary hospital ward.

When TST results were considered separately, the overall prevalence of LTBI among HCWs according to cut-off points of ≥5 mm and ≥10 mm were 60.8% [CI 95%: 57%–64%] and 40% [CI 95%: 36%–43%], respectively. In contrast, only 172 (27%) of 664 HCW tested positive by the QFT [CI 95%: 23%–30%].

Regarding concordance, when the cut-off point of ≥10 mm was chosen, 114 (26%) HCW tested positive by both tests and 322 (74%) tested negative by both tests. When the cut-off used was ≥5 mm, 219 (60.5%) tested negative by both tests and 143 (39.5%) tested positive by both tests. Overall agreement between ≥10 mm and ≥5 mm cut-off points was 69% and 57%, with κ values of 0.31 [CI 95%: 0.24–0.39] and 0.22 [CI 95%: 0.16–0.28], respectively. Indeterminate results of the QuantiFERON-TB Gold In-Tube Test were not reported.

Among the 632 HCW with valid results for both tests, 270 (42.7%) had discordant results for the TST cut-off of ≥5 mm; 241 (89.3%) were TST+/QFT− and 29 (10.7%) were TST−/QFT+. For the cut-off of ≥10 mm TST, 196 (31%) had discordant results, and 138 (70.4%) were TST+/QFT− and 58 (29.6%) were TST−/QFT+. The discordance between the QFT and TST results, assessed by McNemar's test, was significant for both cut-off points (p<0.001).

Next, we investigated possible factors associated with positive TST or QFT results, and discordance of TST vs QFT at the two cut-off points (TST+/QFT− and TST−/QFT+).

By bivariate analysis, a cut-off of ≥5 mm was significantly associated with age (in years) between 36–40 [OR = 1.79; CI 95%: 1.04–3.10], 41–45 [OR = 2.52; CI 95%: 1.41–4.50] and 46–64 [OR = 2.59;CI 95%: 1.60–4.20]; presence of BCG scar [OR = 1,57; CI 95%: 0,99–2,48]; previous TST [OR = 1.44; CI 95%: 1.02–2.04]; smoking [OR = 2.57; CI 95%: 1.28–5.15] and working for greater than 5 years in health care profession at a primary health care setting [OR = 1.7; CI 95%: 1.19–2.44]. By multivariate analysis, age 41–45 [OR = 2.11; CI 95%: 1.13–3.93] and 46–64 [OR = 2.02; CI 95%: 1.14–3.58], and years served in health care profession at primary health care settings [OR = 1.66; CI 95%: 1.12–2.47] remained significant. BCG vaccination scar was also associated with a positive TST result at cut-off of ≥5 mm [OR = 1.78: CI 95%: 1.09–2.90] (Table 1).

**Table 1:** Variables associated with positive results of tuberculin skin test-TST (≥5 mm) health care workers-HCW.

Variables	Test, N	No positive n (%)	Crude OR*	95% CI**	OR* Adjusted	95% CI**
**Gender**						
Male	67	43 (64.18)	Reference		Reference	
Female	565	341 (60.35)	0.84	0.50–1.43	0.70	0.39–1.25
**Age group (years)**						
19–30	101	45 (44.55)	Reference		Reference	
31–35	102	59 (57.84)	1.70	0.97–2.97	1.38	0.77–2.49
36–40	110	65 (59.09)	1.79^a^	**1.04–3.10**	1.60	0.89–2.86
41–45	97	65 (67.01)	2.52^a^	**1.41–4.50**	2.11^a^	**1.13–3.93**
46–64	222	150 (67.57)	2.59^a^	**1.60–4.20**	2.02^a^	**1.14–3.58**
**Presence of BCG scar**						
No	86	44 (51.16)	Reference		Reference	
Yes	546	340 (62.27)	1.57^a^	**0.99–2.48**	1.78^a^	**1.09–2.90**
**Professional category**						
CHW***	302	175 (57.95)	Reference		Reference	
Nurse technician	219	144 (65.75)	1.39	0.97–1.99	0.86	0.45–1.64
Nurse	78	49 (62.82)	1.22	0.73–2.04	0.89	0.40–1.94
Physicians	33	16 (48.48)	0.68	0.33–1.40	0.44	0.16–1.20
**Work only at Primary Health Care**						
No	354	203 (57.34)	Reference		Reference	
Yes	278	181 (65.11)	1.38	1.00–1.91	1.67	0.89–3.13
**Contact with a household member with TB**						
No	501	293 (58.48)	Reference		Reference	
Yes	121	81 (66.94)	1.43	0.94–2.18	1.31	0.84–2.03
Unknown	10	10 (100.00)	Omitted		Omitted	
**Alcohol abuse**						
No	393	229 (58.27)	Reference		Reference	
Yes	239	155 (64.85)	1.32	0.94–1.84	1.19	0.83–1.70
**Prior TST**						
No	420	243 (57.86)	Reference		Reference	
Yes	212	141 (66.51)	1.44^a^	**1.02–2.04**	1.29	0.88–1.88
**Smoker**						
No	475	275 (57.89)	Reference		Reference	
Yes	50	39 (78.00)	2.57^a^	**1.28–5.15**	1.93	0.92–4.02
Ex-smoker	107	70 (65.42)	1.37	0.88–2.13	1.17	0.73–1.88
**Years served in health care profession at primary health care**						
<5	168	86 (51.19)	Reference		Reference	
≥5	464	298 (64.22)	1.71^a^	**1.19–2.44**	1.66^a^	**1.12–2.47**
**Comorbidity**						
No	477	289 (60.59)	Reference		Reference	
Yes	155	95 (61.29)	1.02	0.71–1.49	0.85	0.56–1.28

OR* = Odds ratio;

CI** = Confidence interval;

a = p value<0.05;

CHW*** = community health worker.

By bivariate analysis, a TST cut-off of ≥10 mm showed significant association with age between 41–45 years [OR = 1.90; CI 95%: 1.05–3.45] and 46–64 years [OR = 2.21; CI 95%: 1.33–3.68]; being a nurse technician [OR = 1.66; CI 95%: 1.16–2.36]; having a household contact with TB [OR = 1.81; CI 95%: 1.27–2.57]; prior TST [OR = 1.57; CI 95%: 1.12–2.20]; smoking [OR = 1.94; CI 95%: 1.08–3.48]; and years served in health care profession at primary health care settings [OR = 1.68; CI 95%: 1.16–2.45]. In multivariate analysis, only household contact with TB [OR = 1.89; CI 95%: 1.24–2.87] and years served in health care profession at primary health care settings [OR = 1.77; CI 95%: 1.17–2.67] were associated (Table 2).

**Table 2:** Variables associated with positive results of tuberculin skin test-TST (≥10 mm) in health care workers-HCW.

Variables	Test, N	No positive n (%)	Crude OR*	95% CI**	OR* Adjusted	95% CI**
**Gender**						
Male	67	30 (44,78)	Reference		Reference	
Female	565	222 (39.29)	0.79	0.47–1.32	0.66	0.38–1.17
**Age group (years)**						
19–30	101	28 (27.72)	Reference		Reference	
31–35	102	37 (36.27)	1.48	0.81–2.68	1.19	0.64–2.22
36–40	110	44 (40.0)	1.73	0.97–3.10	1.53	0.83–2.82
41–45	97	41 (42.27)	1.90^a^	**1.05–3.45**	1.37	0.72–2.61
46–64	222	102 (45.95)	2.21^a^	**1.33–3.68**	1.47	0.81–2.65
**Presence of BCG scar**						
No	86	32 (37.21)	Reference		Reference	
Yes	546	220 (40.29)	1.13	0.71–1.82	1.18	0.72–1.95
**Professional category**						
CHW***	302	109 (36.09)	Reference		Reference	
Nurse technician	219	106 (48.40)	1.66^a^	**1.16–2.36**	1.38	0.73–2.61
Nurse	78	29 (37.18)	1.04	0.62–1.75	0.93	0.42–2.05
Physicians	33	8 (24.24)	0.56	0.24–1.29	0.44	0.15–1.28
**Work only at Primary Health Care**						
No	354	130 (36.72)	Reference		Reference	
Yes	278	122 (43.88)	1.34	0.97–1.85	1.25	0.67–2.34
**Contact with a household member with TB**						
No	501	182 (36.33)	Reference		Reference	
Yes	121	65 (53.72)	1.81^a^	**1.27–2.57**	1.89^a^	**1.24–2.87**
Unknown	10	5 (50.0)	1.75	0.50–6.13	1.61	0.43–6.06
**Alcohol abuse**						
No	393	149 (37.91)	Reference		Reference	
Yes	239	103 (43.10)	1.24	0.89–1.72	1.17	0.82–1.67
**Prior TST**						
No	420	152 (36.19)	Reference		Reference	
Yes	212	100 (47.17)	1.57^a^	**1.12–2.20**	1.41	0.97–2.03
**Smoker**						
No	475	179 (37.68)	Reference		Reference	
Yes	50	27 (54.0)	1.94^a^	**1.08–3.48**	1.52	0.81–2.86
Ex-smoker	107	46 (42.99)	1.24	0.81–1.90	0.99	0.62–1.57
**Years served in health care profession at primary health care**						
<5	168	52 (30.95)	Reference		Reference	
≥5	464	200 (43.10)	1.68^a^	**1.16–2.45**	1.77^a^	**1.17–2.67**
**Comorbidity**						
No	477	187 (39.20)	Reference		Reference	
Yes	155	65 (41.94)	1.12	0.77–1.61	0.95	0.64–1.43

OR* = Odds ratio;

CI** = Confidence interval;

a = p value<0.05;

CHW*** = community health worker.

By bivariate analysis, positive QFT result was statistically significantly associated with age between 46–64 years [OR = 2.40; CI 95%: 1.34–4.28]; household contact with TB [OR = 2.03; CI 95%: 1.36–3.03]; smoking [OR = 2.16; CI 95%: 1.19–3.94]; years served in health care profession at primary health care settings [OR = 1.75; CI 95%: 1.14–2.68]; and comorbidity [OR = 1.50; CI 95%: 1.01–2.22]. The presence of BCG scar was statistically significant with a lower probability of having a positive QFT result [OR = 0.55; CI 95%: 0.34–0.88]. The female gender showed a lower probability of testing positive by QFT [OR = 0.59; CI 95%: 0.35–1.00]. In multivariable analysis, years served in health care profession at primary health care settings [OR = 1.70; CI 95%: 1.07–2.71] remained statistically significant (Table 2). The presence of BCG scar [OR = 0.54; CI 95%: 0.32–0.90] and female gender [OR = 0.47; CI 95%: 0.26–0.85] remained less likely to be positive by QFT (Table 3).

**Table 3:** Variables associated with positive test results with the Quantiferon TB Gold in tube test - QFT in health care workers - HCW.

Variables	Test, N	No positive n (%)	Crude OR*	95% CI**	OR* Adjusted	95% CI**
**Gender**						
Male	67	25 (37.31)	Reference		Reference	
Female	565	147 (26.02)	0.59^a^	0.35–1.00	0.47^a^	**0.26–0.85**
**Age group (years)**						
19–30	101	18 (17.82)	Reference		Reference	
31–35	102	23 (22.55)	1.34	0.67–2.67	1.19	0.58–2.45
36–40	110	31 (28.18)	1.80	0.94–3.50	1.63	0.81–3.27
41–45	97	24 (24.74)	1.52	0.76–3.01	1.26	0.61–2.64
46–64	222	76 (34.23)	2.40^a^	**1.34–4.28**	1.90	0.97–3.70
**Presence of BCG scar**						
No	86	33 (38.37)	Reference		Reference	
Yes	546	139 (25.46)	0.55^a^	**0.34–0.88**	0.54^a^	**0.32–0.90**
**Professional category**						
CHW***	302	78 (25.83)	Reference		Reference	
Nurse technician	219	69 (31.51)	1.32	0.90–1.94	1.37	0.68–2.74
Nurse	78	21 (26.92)	1.06	0.60–1.85	1.42	0.61–3.34
Physicians	33	4 (12.12)	0.40	0.13–1.16	0.32	0.08–1.22
**Work only at Primary Health Care**						
No	354	93 (26.27)	Reference		Reference	
Yes	278	79 (28.42)	1.35	0.98–1.85	0.94	0.48–1.85
**Contact with a household member with TB**						
No	501	130 (25.95)	Reference		Reference	
Yes	121	40 (33.06)	2.03	1.36–3.03	1.31	0.83–2.06
Unknown	10	2 (20.0)	1.75	0.50–6.13	0.47	0.09–2.37
**Alcohol abuse**						
No	393	102 (25.95)	Reference		Reference	
Yes	239	70 (29.29)	1.18	0.82–1.70	1.17	0.79–1.72
**Prior TST**						
No	420	109 (25.95)	Reference		1	
Yes	212	63 (29.72)	1.20	0.83–1.74	1.00	0.67–1.50
**Smoker**						
No	475	119 (25.05)	Reference		Reference	
Yes	50	21 (42.00)	2.16^a^	**1.19–3.94**	1.77	0.93–3.36
Ex-smoker	107	32 (29.91)	1.27	0.80–2.02	0.98	0.60–1.62
**Years served in health care profession at primary health care**						
<5	168	33 (19.64)	Reference		Reference	
≥5	464	139 (29.96)	1.75^a^	**1.14–2.68**	1.70^a^	**1.07–2.71**
**Comorbidity**						
No	477	120 (25.16)	Reference		Reference	
Yes	155	52 (33.55)	1.50^a^	**1.01–2.22**	1.31	0.85–2.00

OR* = Odds ratio;

CI** = Confidence interval;

a = p value<0.05;

CHW*** = community health worker.

Variables to determine the factors associated with discordance between the TST for both cut-offs and QFT are shown in Tables 4 and 5.

**Table 4:** Variables associated with discordant groups (TST+/QFT− and TST−/QFT+) compared to the reference group (TST−/QFT−) at TST cut-off ≥5 mm.

	TST+/QFT− (≥5 mm)						TST−/QFT+ (≥5 mm)					
Variables	Test, N	N (%)	Crude OR*	95% CI**	OR* Adjusted	95% CI**	Test, N	N (%)	Crude OR*	95% CI**	OR* Adjusted	95% CI**
**Gender**												
Male	42	22 (52,38)	Reference		Reference		23	3 (13.04)	Reference		Reference	
Female	417	219 (52,52)	1	0.53–1.89	0.82	0.39–1.68	224	26 (11.61)	0.87	0.24–3.15	0.63	0.14–2.74
**Age group (years)**												
19–30	83	31 (37.35)	Reference		Reference		56	4 (7.14)	Reference		Reference	
31–35	79	40 (50.63)	1.72	0.91–3.21	1.55	0.79–3.05	43	4 (9.30)	1.33	0.31–5.66	1.58	0.33–7.52
36–40	79	40 (50.63)	1.72	0.91–3.21	1.74	0.89–3.38	45	6 (13.33)	2.00	0.52–7.57	2.15	0.48–9.65
41–45	73	45 (61.64)	2.69^a^	**1.40–5.15**	2.70^a^	**1.32–5.51**	31	3 (9.68)	1.39	0.29–6.66	1.40	0.25–7.81
46–64	145	85 (58.62)	2.37^a^	**1.36–4.13**	2.04^a^	**1.05–3.93**	72	12 (16.67)	2.6	0.79–8.55	2.26	0.52–9.68
**Presence of BCG scar**												
No	53	19 (35.85)	Reference		Reference		42	8 (19.05)	Reference		Reference	
Yes	406	222 (54.68)	2.15^a^	**1.19–3.91**	2.72^a^	**1.40–5.25**	205	21 (10.24)	0.48	0.19–1.18	0.43	0.16–1.18
**Professional category**												
CHW***	224	106 (47.32)	Reference		Reference		127	9 (7.09)	Reference		Reference	
Nurse technician	150	89 (59.33)	1.62^a^	**1.06–2.46**	0.77	0.36–1.64	74	13 (17.57)	2.79^a^	**1.13–6.90**	2.68	0.57–12.51
Nurse	56	33 (58.93)	1.59	0.88–2.89	0.77	0.30–2.00	29	6 (20.69)	3.42^a^	**1.10–10.54**	5.57	0.93–33.21
Physicians	29	13 (44.83)	0.90	0.41–1.96	0.46	0.15–1.39	17	1 (5.88)	0.81	0.09–6.90	0.82	0.05–11.32
**Work only at Primary Health Care**												
No	261	122 (46.74)	Reference		Reference		151	12 (7.95)	Reference		Reference	
Yes	198	119 (60.10)	1.71^a^	**1.18–2.49**	2.30^a^	**1.09–4.86**	96	17 (17.71)	2.49^a^	**1.13–5.48**	1.04	0.24–4.45
**Contact with a household member with TB**												
No	371	190 (51.21)	Reference		Reference		207	26 (12.56)	Reference		Reference	
Yes	80	43 (53.75)	1.10	0.68–1.79	0.98	0.58–1.65	40	3 (7.50)	0.56	0.16–1.96	0.53	0.13–2.07
Unknown	8	8 (100)	Omitted	Omitted	Omitted	Omitted	0	0	Omitted	Omitted	Omitted	Omitted
**Alcohol abuse**												
No	290	143 (49.31)	Reference		Reference		163	16 (9.82)	Reference		Reference	
Yes	169	98 (57.99)	1.41	0.96–2.07	1.28	0.85–1.94	84	13 (15.48)	1.68	0.76–3.68	2.04	0.83–5.03
**Prior TST**												
No	311	155 (49.84)	Reference		Reference		177	21 (11.86)	Reference		Reference	
Yes	148	86 (58.11)	1.39	0.94–2.07	1.20	0.77–1.87	70	8 (11.43)	0.95	0.40–2.27	0.58	0.21–1.59
**Smoker**												
No	355	178 (50.14)	Reference		Reference		199	22 (11.06)	Reference		Reference	
Yes	29	20 (68.97)	2.20	0.97–4.98	1.79	0.74–4.32	11	2 (18.18)	1.78	0.36–8.81	1.05	0.18–6.02
Ex-smoker	75	43 (57.33)	1.33	0.80–2.20	1.14	0.66–1.98	37	5 (13.51)	1.25	0.44–3.56	0.93	0.26–3.23
**Years served in health care profession at primary health care**												
<5	135	61 (45.19)	Reference		Reference		82	8 (9.76)	Reference		Reference	
≥5	324	180 (55.56)	1.51^a^	**1.01–2.27**	1.41	0.89–2.23	165	21 (12.73)	1.34	0.57–3.19	1.48	0.58–3.76
**Comorbidity**												
No	356	188 (52.81)	Reference		Reference		187	19 (10.16)	Reference		Reference	
Yes	103	53 (51.46)	0.94	0.61–1.46	0.82	0.50–1.33	60	10 (16.67)	1.76	0.77–4.04	1.98	0.75–5.19

OR* = Odds ratio;

CI** = Confidence interval;

a = p value<0.05;

CHW*** = community health worker.

**Table 5:** Variables associated with discordant groups (TST+/QFT− and TST−/QFT+) compared to the reference group (TST−/QFT−) at TST cut-off ≥10 mm.

	TST+/QFT− (≥10 mm)						TST−/QFT+ (≥10 mm)					
Variables	Test, N	N (%)	Crude OR*	95% CI**	OR* Adjusted	95% CI**	Test, N	N (%)	Crude OR*	95% CI**	OR* Adjusted	95% CI**
**Gender**												
Male	42	9 (21.43)	Reference		Reference		37	4 (10.81)	Reference		Reference	
Female	418	130 (31.10)	1.65	0.76–3.55	1.46	0.63–3.37	342	54 (15.79)	1.54	0.52–4.54	1.14	0.36–3.65
**Age group (years)**												
19–30	83	16 (19.28)	Reference		Reference		73	6 (8.22)	Reference		Reference	
31–35	79	23 (29.11)	1.71	0.82–3.56	1.50	0.69–3.24	65	9 (13.85)	1.79	0.60–5.34	1.74	0.56–5.40
36–40	79	23 (29.11)	1.71	0.82–3.56	1.68	0.78–3.61	66	10 (15.15)	1.99	0.68–5.82	1.81	0.59–5.57
41–45	73	26 (35.62)	2.31^a^	**1.12–4.78**	1.80	0.82–3.97	56	9 (16.07)	2.13	0.71–6.41	1.86	0.57–5.99
46–64	146	51 (34.93)	2.24^a^	**1.18–4.27**	1.60	0.76–3.36	119	24 (20.17)	2.82^a^	**1.09–7.27**	2.36	0.79–7.00
**Presence of BCG scar**												
No	53	9 (16.98)	Reference		Reference		54	10 (18.52)	Reference		Reference	
Yes	407	130 (31.94)	2.29^a^	**1.08–4.84**	2.26^a^	**1.03–4.91**	325	48 (14.77)	0.76	0.35–1.61	0.69	0.31–1.54
**Professional category**												
CHW***	224	59 (26.34)	Reference		Reference		192	27 (14.06)	Reference		Reference	
Nurse technician	150	57 (38)	1.71^a^	**1.09–2.67**	0.76	0.33–1.73	113	20 (17.70)	1.31	0.69–2.47	0.74	0.22–2.51
Nurse	57	17 (29.82)	1.18	0.62–2.25	0.49	0.17–1.40	49	9 (18.37)	1.37	0.59–3.15	1.00	0.24–4.20
Physicians	29	6 (20.69)	0.72	0.28–1.87	0.38	0.10–1.39	25	2 (8.00)	0.53	0.11–2.38	0.30	0.04–2.21
**Work only at Primary Health Care**												
No	261	68 (26.05)	Reference		Reference		223	30 (13.45)	Reference		Reference	
Yes	199	71 (35.68)	1.57^a^	**1.05–2.35**	2.21	0.97–4.99	156	28 (17.95)	1.40	0.80–2.46	1.62	0.49–5.31
**Contact with a household member with TB**												
No	371	102 (27.49)	Reference		Reference		319	50 (15.67)	Reference		Reference	
Yes	81	34 (41.98)	1.90^a^	**1.16–3.13**	1.72^a^	**1.01–2.92**	55	8 (14.55)	0.91	0.40–2.05	0.83	0.35–1.93
Unknown	8	3 (37.50)	1.58	0.37–6.74	2.29	0.48–10.91	5	0 (0)	Omitted	Omitted	Omitted	Omitted
**Alcohol abuse**												
No	291	79 (27.15)	Reference		Reference		243	31 (12.76)	Reference		Reference	
Yes	169	60 (35.50)	1.47	0.98–2.22	1.42	0.92–2.20	136	27 (19.85)	1.69	0.96–2.98	1.68	0.91–3.07
**Prior TST**												
No	311	80 (25.72)	Reference		Reference		267	36 (13.48)	Reference		Reference	
Yes	149	59 (39.60)	1.89^a^	**1.24–2.86**	1.66^a^	**1.05–2.62**	112	22 (19.64)	1.56	0.87–2.81	1.21	0.64–2.30
**Smoker**												
No	356	100 (28.09)	Reference		Reference		296	40 (13.51)	Reference		Reference	
Yes	29	11 (37.93)	1.56	0.71–3.42	1.14	0.48–2.72	23	5 (21.74)	1.77	0.62–5.05	1.42	0.46–4.34
Ex-smoker	75	28 (37.33)	1.52	0.90–2.57	1.18	0.67–2.09	60	13 (21.67	1.77	0.88–3.56	1.38	0.64–2.94
**Years served in health care profession at primary health care**												
<5	135	35 (25.93)	Reference		Reference		116	16 (13.79)	Reference		Reference	
≥5	325	104 (32.0)	1.34	0.85–2.10	1.26	0.77–2.08	263	42 (15.97)	1.18	0.63–2.21	1.01	0.51–1.98
**Comorbidity**												
No	357	108 (30.25)	Reference		Reference		289	40 (13.84)	Reference		Reference	
Yes	103	31 (30.10)	0.99	0.61–1.60	0.82	0.48–1.39	90	18 (20.0)	1.55	0.84–2.87	1.32	0.67–2.60

OR* = Odds ratio;

CI** = Confidence interval;

a = p value<0.05;

CHW*** = community health worker.

At a cut-off of ≥5 mm, the multivariate test showed that the group with TST+/QFT− discordance was more likely to be between the age 41–45 years [OR = 2.70; CI 95%: 1.32–5.51], 46–64 years [OR = 2.04; CI 95%: 1.05–3.93], have a BCG scar [OR = 2.72; CI 95%: 1.40–5.25] and worked only in primary health care [OR = 2.30; CI 95%: 1.09–4.86]. The group with discordance of TST−/QFT+ revealed no statistically significant association with any of the variables evaluated (Table 4).

At a cut-off of ≥10 mm, the group with discordance of TST+/QFT− was significantly likely to have a BCG scar [OR = 2.26; CI 95%: 1.03–4.91], being a household contact of a TB patient [OR = 1.72; CI 95%: 1.01–2.92] and having had a previous TST [OR = 1.66; CI 95%: 1.05–2.62]. The TST−/QFT+ group showed no statistically significant association with any of the variables (Table 5).

We compared the discordant results (TST+/QFT− and TST−/QFT+) depending on the IFN-γ concentration at both cut-offs of TST (Table 6). Among the TST−/IGRA+ group, 29 (12%) and 58 (15%) of HCWs, respectively, had a TST cut-off of ≥5 mm and ≥10 mm. Of these, 7 (24.14%) and 10 (17.24%), respectively had borderline IFN-γ concentration (0.2–0.5 IU/ml).

**Table 6:** Comparing the discordant results (TST+/QFT− and TST−/QFT+) depending on the categorized IFN-γ concentration (IU/mL).

IFN-γ concentration (IU/mL)	≥5 mm		*P* value*	≥10 mm		*P* value*
	TST+/QFT−	TST−/QFT+		TST+/QFT−	TST−/QFT+	
<0.2	216 (100)	0 (0)		121 (100)	0 (0)	
0.2–0.5	24 (77.42)	7 (22.58)		17 (62.96)	10 (37.04)	
>0.5	1 (4.35)	22 (95.65)	<0.001	1 (2.04)	48 (97.96)	<0.001

*Fisher's exact test.

## Discussion

Our study shows that the prevalence of LTBI among HCW in primary HCW in four state capitals of Brazil varied according to definition based on different TST cut-off points. The prevalence at ≥5 mm and ≥10 mm cut-off, was 60.8% and 40% respectively, and 27% if QFT was used. Despite these differences, the prevalence among HCW was higher than the estimated prevalence of LTBI among the Brazilian general population [1]. These findings are consistent with other studies carried out in Brazil. A prospective cohort study was performed by Moreira et al (2010) in Brazil, which estimated that among community health workers involved in disease control, the incidence of positive TST reaction during the follow-up was 41.7% in those exposed to patients with active tuberculosis [11].

Regarding the high prevalence of positive TST or IGRA among Brazilian primary HCW, it is important to highlight that the screening of HCW for LTBI is not fully implemented in healthcare facilities in Brazil. Less than 33% had previously received the tuberculin test.

Our study had some limitations: first, there is no “gold standard” for detecting LTBI, and second, we did not evaluate a possible booster effect of TST, although the Brazilian TST guideline does not require assessing booster effect on HCW [18]. Nevertheless, the strength of the present study was the large sample size and its multi-regional survey design.

In previous studies, several risk factors have been associated with high prevalence of positive TST and QFT [11], [19], [20]. Although older age has been reported to be associated with positive results with both tests [21], [22], our study found this association only with the cut-off of ≥5 mm. Household contact with index cases of TB was associated with a positive TST at a cut-off of ≥10 mm, which was also shown by other studies [19], [21], [22].

Another associated factor was occupation or working as HCW for more than five years, which was significantly associated with positive TST results regardless of TST cut-offs, as well as with QFT. This finding concurs with other reports [19], [23]–[25]. We also found that being a female is associated with a lower prevalence of LTBI when QFT is used, also reported by another study [26]. The reason for this association is unclear.

The observed concordance between TST (≥5 mm and ≥10 mm) and QFT results was lower than that reported by Pai et al. (2005) (k = 0.61) among health professionals in India, a country endemic for TB also with a high BCG vaccination coverage [27]. Studies by other research groups have found low concordance between the same two TST cut-offs with QFT [28], [29], [30], [31].

In this study, TST+/QFT− discordance was more frequent than TST−/QFT+ discordance.

We summarized all the risk factors significantly associated with various combinations of TST and QFT results (Table 7). In a study carried out in Salvador-Brazil with household TB contacts that was based on TST cut-off of ≥10 mm, the discordant subgroup TST+/QFT− shared characteristics similar to those observed in the concordant group TST+/QFT+ instead of the TST−/QFT− group [32]. In our study, the TST+/QFT− group, at the cut-off of ≥5 mm TST, was older, had a BCG scar, and worked only in the primary health care. At the cut-off of ≥10 mm, the TST+/QFT− group was more likely to have a BCG scar, be a household contact of TB, and had TST done previously.

**Table 7:** Summary table containing all the risk factors found to be significantly associated with various combinations of tuberculin skin test (TST) and QFT results.

TST/QFT	QFT+	OR* Adjusted	CI **95%	QFT−	OR* Adjusted	CI** 95%
	Age 46–64	3.28	1.06–4.42	Age 41–45	2.70	1.32–5.51
**TST+ (≥5 mm)**	Smoker	2.97	1.17–7.50	Age 46–64	2.04	1.05–3.93
	Years served in health care profession at primary health care >5 years	2.17	1.06–4.42	Presence of BCG scar	2.72	1.40–5.25
				Worked only in primary health care	2.30	1.09–4.86
**TST+ (≥10 mm)**	**Contact with a household member with TB**	2.14	1.22–3.75	Presence of BCG scar	2.26	1.03–4.91
	Years served in health care profession at primary health care >5 years	2.70	1.29–5.65	**Contact with a household member with TB**	1.72	1.01–2.92
				Prior TST	1.66	1.05–2.62
**TST− (≥5 mm)**	no variables statistically significant			Reference group		
**TST− (≥10 mm)**	no variables statistically significant			Reference group		

OR* = Odds ratio;

CI** = Confidence interval.

A study carried out in the United States among HCWs with increased risk of LTBI discusses the possibility of TST+/QFT− discordance to represent an exposure to *M. tuberculosi*s in the remote past [33]. Our study and the study in American HCWs do not show any data that might suggest that TST+/IGRA− negative results might be caused by remote infection. The discordant TST+/QFT− result is more consistent with strong association of BCG with positive TST [34]. However, our observation differs from the result of the study of household contacts in Salvador-Brazil, which did not show an association with BCG vaccination with TST cut-off of ≥10 mm [32].

On the other hand, the TST−/IGRA+ result, observed in 29 (12%) and 58 (15%) of HCWs, respectively when a cut-off point of ≥5 mm and ≥10 mm was used, was suggested to be associated with recent exposure [35]. In our study, the subgroup with TST−/QFT+ results at both cut-offs showed QFT ELISA values that were borderline between 0.2 and 0.5 IU/ml [36]. These values may represent false-positive QFT results.

It should be noted however that, although TST+/QFT− and TST−/QFT+ discordant results suggest previous exposure (recent or remote) to *M. tuberculosis*, neither TST nor QFT can distinguish remote from recent infection [37].

A study by Pai and colleagues (2009) concluded that health professionals should be cautious about using a simplistic dichotomous criterion to determine conversion or reversion, and should instead consider the amount of change in absolute IFN-γ responses, as well as relevant clinical information to interpret serial testing results [36].

Several guidelines have sought to standardize the use of IGRA and TST, according to the characteristic of each country and specific population groups [38]–[40]. In relation to HCW, there are guidelines that define the use of one or the other test to diagnose LTBI. In many high-income countries with low rates of TB, serial testing for LTBI is recommended for persons at increased risk of TB [41]. The advantages are to increase the test specificity in individuals with prior BCG vaccination, and also to reduce cost incurred by the follow-up and treatment of LTBI based on false-positive TST. However, the use of IGRAs for serial testing is complicated by the lack of clear data on optimal cut-offs for serial testing and unclear interpretation and prognosis of conversions and reversions, reproducibility or time interval to conversion of IGRA after exposure to tuberculosis [20], [40], [42], [43]. Some studies have shown considerable fluctuations in positive and negative IGRA results from the same individuals [44], [45], [46].

These discussions around the limitations, advantages and applicability of QFT in clinical practice are important, especially in low and middle income countries with high incidence of tuberculosis, BCG vaccination coverage and the presence of environmental mycobacteria. In our study among Brazilian HCW, we found high positive results by TST, using different TST cut-offs points, ≥5 mm and ≥10 mm, and QFT. However, we found a high level of disagreement with QFT, regardless of the TST cut-off. Although we identified that BCG vaccination may partly account for this disagreement, we suggest that Brazilian recommendations to treat LTBI, based on information gathered from medical history, TST, chest radiograph and physical examination, should not be changed. Further studies are needed before the QFT is introduced in prospective LTBI screening program for HCWs in Brazil.

## Supporting Information

Questionnaire S1 **Screening of community health workers.** Questionnaire to identify the personal characteristics of community health workers and the level of exposure to *Mycobacterium tuberculosis* (in portuguese).
(PDF)

Questionnaire S2 **Screening of health care workers.** Questionnaire to identify the personal characteristics of nursing technicians, nurses and physicians and the level of exposure to *Mycobacterium tuberculosis* (in portuguese).
(PDF)
