# Supplementary material for: Comparison of Interferon-γ Release Assay to Two Cut-Off Points of Tuberculin Skin Test to Detect Latent Mycobacterium tuberculosis Infection in Primary Health Care Workers
Source: PLoS One. 2014 Aug 19;9(8):e102773. doi: 10.1371/journal.pone.0102773 (PMC4138087; doi:10.1371/journal.pone.0102773)
Supplement: Questionnaire S1 — Screening of community health workers. Questionnaire to identify the personal characteristics of community health workers and the level of exposure to Mycobacterium tuberculosis (in portuguese). (PDF) [file pone.0102773.s001.pdf]

12021

PROJETO INATA - INFECÇÃO E ADOECIMENTO POR TUBERCULOSE ENTRE PROFISSIONAIS DE SAÚDE DA ATENÇÃO BÁSICA

Triagem de Agentes Comunitários de Saúde

Este formulário deve ser preenchido para **AGENTES COMUNITÁRIOS DE SAÚDE**. O objetivo deste questionário é descrever as características pessoais de cada profissional e o nível de exposição ao *Mycobacterium tuberculosis*

1. Identificação: ☐☐☐ - ☐☐☐☐ (iniciais - número do estudo)

2. Data da avaliação: ☐☐ / ☐☐ / ☐☐☐☐ (DD / MM / AAAA)

3.Município: ☐

4. Estado: ☐☐

5. Tipo de Unidade que o profissional trabalha:
- ☐ Unidade de saúde da família
- ☐ Unidade Básica com PACS
- ☐ Outro. Especificar: ☐

6. Nesta Unidade há Programa de Controle de Tuberculose implantado?
☐ Sim ☐ Não ☐ Não sabe

DADOS DEMOGRÁFICOS

7. Sexo: ☐ Masculino ☐ Feminino

8. Data de nascimento: ☐☐ / ☐☐ / ☐☐☐☐ (DD / MM / AAAA)

DADOS OCUPACIONAIS

9. Formação profissional:
- ☐ Nível fundamental
- ☐ Nível médio
- ☐ Nível técnico
- ☐ Nível universitário

10. Tempo que trabalha na função na rede básica do município: ☐☐ ☐ anos ☐ meses

11. Você já conviveu com alguma pessoa com TB?
- ☐ Sim. Quem? ☐
- ☐ Não
- ☐ Não sabe

12. Você já trabalhou em ambiente com alto risco de exposição para tuberculose?
- ☐ Sim. Onde? ☐
- ☐ Não
- ☐ Não sabe

13. Com que periodicidade você realiza visitas domiciliares?
- ☐ Diariamente
- ☐ 3 vezes por semana
- ☐ 2 vezes por semana
- ☐ 1 vez por semana
- ☐ Outro. Especificar: ☐

14. Qual a média de tempo que você permanece em cada domicílio? ☐☐☐ minutos

15. Você realiza visitas em casa de repouso ou sistema prisional?
- ☐ Sim. Por quanto tempo? ☐☐ ☐ anos ☐ meses
- ☐ Não

16. Com que frequência você suspeita que um usuário que apresenta tosse pode ser doente de TB?
- ☐ Nunca
- ☐ Quase nunca
- ☐ As vezes
- ☐ Quase sempre
- ☐ Sempre

17. Qual o número de doentes de TB que você já acompanhou?

17a. Desde que iniciou seu trabalho como ACS? ☐☐

17b. Nos últimos 3 anos? ☐☐

18. Você já supervisionou um tratamento para tb?

☐ Sim ☐ Não

18a. Em quantos pacientes? ☐☐

18b. Com qual periodicidade foram as supervisões?

☐ Diariamente
☐ 3 vezes por semana
☐ 2 vezes por semana
☐ 1 vez por semana

19. Com que frequência há equipamento de proteção individual disponível na Unidade de Saúde?

☐ Nunca (vá para a questão 20)
☐ Quase nunca
☐ As vezes
☐ Quase sempre
☐ Sempre

19a. O Equipamento de Proteção individual é de fácil acesso?

☐ Sim ☐ Não

20. Quando você está com um paciente sintomático respiratório, com que frequência você usa equipamento de proteção individual?

☐ Nunca
☐ Quase nunca
☐ As vezes
☐ Quase sempre
☐ Sempre

21. Durante o atendimento de sintomático respiratório, em que momento você utiliza proteção respiratória?

☐ Antes de entrar no domicílio
☐ Ao entrar no domicílio
☐ Durante a visita
☐ Ao encontrar o doente de TB
☐ Não utuliza
☐ Outro. Especificar: ☐

22. Você já recebeu algum treinamento ou capacitação sobre TB?

☐ No último ano
☐ 1 a 2 anos
☐ 3 a 4 anos
☐ 5 anos ou mais
☐ Não fez (vá para a pergunta 23)

22a. Como você avalia a contribuição do(s) treinamento(s) para a sua prática clínica?

☐ Muito boa
☐ Boa
☐ Regular
☐ Ruim
☐ Muito ruim

23. Com que frequência você busca informações sobre TB?

☐ Nunca
☐ Quase nunca
☐ As vezes
☐ Quase sempre
☐ Sempre

AVALIAÇÃO DO AMBIENTE

24. A recepção da Unidade possui ventilação adequada?(fluxo de ar)

☐ Sim ☐ Não

25. Número de janelas/similares que ficam abertas diariamente para ventilação do ambiente: ☐☐

26. Número de portas/similares que ficam abertas diariamente para ventilação do ambiente: ☐☐

FATORES DE RISCO

27. Você possui alguma comorbidade ou faz uso de alguma droga imunossupressora?

☐ Sim ☐ Não

27a. Se sim, qual?

☐

12021

☐☐☐ - ☐☐☐☐
iniciais número do estudo

FATORES DE RISCO

34. Você alguma vez fumou?

☐ Sim

☐ Não (vá para a questão 35)

34a. Se sim, fuma agora?

☐ Sim ☐ Não

34b. Anos que fumou ☐☐

34c. Número médio de cigarros/dia ☐☐

34. Você alguma ingeriu bebida alcóolica?

☐ Sim

☐ Não (vá para a questão 36)

35a. Se sim, bebe agora?

☐ Sim ☐ Não

35b. Anos que bebeu ☐☐

35c. Se sim, frequência que bebe:

☐ Raramente (,1dia/semana)

☐ Ocasionalmente (1-3 dias/semana)

☐ Frequentemente (4 a 6 dias/semana)

☐ Diariamente

☐ Recusou responder

HISTÓRIA ANTERIOR RELACIONADA A TB

36. Você já realizou teste tuberculínico?

☐ Sim. Quando? ☐☐ / ☐☐ / ☐☐☐☐ (DD / MM / AAAA)

☐ Não

☐ Não sabe

36a. Resultado do teste tuberculínico: ☐☐ mm

37. Você já recebeu terapia preventiva de TB?

☐ Sim ☐ Não ☐ Não sabe

38. Você está em tratamento ou já tratou TB?

☐ Sim ☐ Não

INVESTIGAÇÃO

39. A cicatriz de BCG está presente?

☐ Sim ☐ Não ☐ Incerta

40.O resultado do Raio X é suspeito para TB?

☐ Sim ☐ Não ☐ Incerto

41. O teste HIV foi realizado?

☐ Sim ☐ Não ☐ Recusa

41a. Resultado do teste HIV:

☐ Negativo

☐ Positivo

☐ Indeterminado

42A coleta de sangue foi realizada?

☐ Sim ☐ Não ☐ Recusa

43. Comentários:

PARA USO DO COLETOR DE DADOS

Iniciais ☐☐☐

Data da coleta: ☐☐ / ☐☐ / ☐☐☐☐ (DD / MM / AAAA)

Assinatura:

PARA USO DO DIGITADOR

Iniciais ☐☐☐

Data do registro: ☐☐ / ☐☐ / ☐☐☐☐ (DD / MM / AAAA)
