# Supplementary material for: Comparison of Interferon-γ Release Assay to Two Cut-Off Points of Tuberculin Skin Test to Detect Latent Mycobacterium tuberculosis Infection in Primary Health Care Workers
Source: PLoS One. 2014 Aug 19;9(8):e102773. doi: 10.1371/journal.pone.0102773 (PMC4138087; doi:10.1371/journal.pone.0102773)
Supplement: Questionnaire S2 — Screening of health care workers. Questionnaire to identify the personal characteristics of nursing technicians, nurses and physicians and the level of exposure to Mycobacterium tuberculosis (in portuguese). (PDF) [file pone.0102773.s002.pdf]

17740

PROJETO INATA - INFECÇÃO E ADOECIMENTO POR TUBERCULOSE ENTRE PROFISSIONAIS DE SAÚDE DA ATENÇÃO BÁSICA

Triagem de Médicos /Enfermagem

Este formulário deve ser preenchido para médicos, enfermeiros e auxiliares/técnicos de enfermagem. O objetivo deste questionário é descrever as características pessoais de cada profissional e o nível de exposição ao Mycobacterium tuberculosis

1. Identificação: ☐☐☐ - ☐☐☐☐ (iniciais / número do estudo)

2. Data da avaliação: ☐☐ / ☐☐ / ☐☐☐☐ (DD / MM / AAAA)

3.Município: ☐

4. Estado: ☐☐

5. Tipo de Unidade que o profissional trabalha:
- ☐ Unidade de saúde da família
- ☐ Unidade Básica de saúde
- ☐ Unidade Básica com PACS
- ☐ Pronto atendimento
- ☐ Outro. Especificar: ☐

6. Nesta Unidade há Programa de Controle de Tuberculose implantado?
☐ Sim ☐ Não ☐ Não sabe

7. Você atualmente trabalha em Programa de Controle de Tuberulose?
☐ Sim ☐ Não

DADOS DEMOGRÁFICOS

8. Sexo: ☐ Masculino ☐ Feminino

9. Data de nascimento: ☐☐ / ☐☐ / ☐☐☐☐ (DD / MM / AAAA)

DADOS OCUPACIONAIS

10. Formação profissional:
- ☐ Médico
- ☐ Enfermeiro
- ☐ Auxiliar/técnico

11. Tempo de formação: ☐☐ anos

12.Possui alguma especialidade? ☐

13.Outra formação profissional? ☐

14. Função/cargo que exerce na Unidade de Saúde neste momento: ☐

15. Número de anos que trabalha na função na rede básica do município: ☐☐ ☐ anos ☐ meses

16. Quais os vínculos que você já teve/tem no serviço público? Por quanto tempo?

	ANOS	MESES
☐ Pronto atendimento	☐☐	☐☐
☐ Ambulatório	☐☐	☐☐
☐ Clínica especialidades	☐☐	☐☐
☐ Hospital (especificar)	☐☐	☐☐
☐ Outro (especificar)	☐☐	☐☐

☐

17. Quais os vínculos que você já teve/tem no serviço privado? Por quanto tempo?

	ANOS	MESES
☐ Pronto atendimento	☐☐	☐☐
☐ Ambulatório	☐☐	☐☐
☐ Clínica especialidades	☐☐	☐☐
☐ Hospital (especificar)	☐☐	☐☐
☐ Outro (especificar)	☐☐	☐☐

☐

18. Qual é a sua principal atividade na Unidade de Saúde? (Marque quantas aplicar)
- ☐ Consulta clínica
- ☐ Visita domiciliar
- ☐ Serviço administrativo
- ☐ Acolhimento/triagem
- ☐ Laboratório
- ☐ Imunização
- ☐ Sala Medicação
- ☐ Expurgo
- ☐ Outro. Especificar: ☐

19. Você já conviveu com alguém com TB?

- ☐ Sim. Quem?
- ☐ Não
- ☐ Não sabe

20. Você já trabalhou em ambiente com alto risco de exposição ao M. tuberculosis? (Necropsia, laboratório, RX, enfermaria de pneumologia)

- ☐ Sim. Onde?
- ☐ Não
- ☐ Não sabe

21. Você já trabalhou em casa de repouso/ asilo ou sistema prisional?

- ☐ Sim. Por quanto tempo? anos
- ☐ Não

22. Você atende ou já atendeu paciente com TB?

☐ Sim ☐ Não

23. Com que frequência há equipamento de proteção individual disponível na Unidade de Saúde?

- ☐ Nunca (vá para a questão 25)
- ☐ Quase nunca
- ☐ As vezes
- ☐ Quase sempre
- ☐ Sempre

24. O Equipamento de Proteção individual é de fácil acesso?

☐ Sim ☐ Não

25. Quando você está com um paciente sintomático respiratório, com que frequência você usa equipamento de proteção individual?

- ☐ Nunca
- ☐ Quase nunca
- ☐ As vezes
- ☐ Quase sempre
- ☐ Sempre

26. Durante o atendimento de sintomático respiratório, em que momento você utiliza proteção respiratória?

- ☐ Antes de iniciar o tratamento
- ☐ Durante o atendimento
- ☐ Durante o exame físico
- ☐ Não utiliza
- ☐ Outro. Especificar:

27. Você já recebeu algum treinamento ou capacitação sobre TB?

- ☐ No último ano
- ☐ 1 a 2 anos
- ☐ 3 a 4 anos
- ☐ 5 anos ou mais
- ☐ Não fez (vá para a pergunta 28)

27a. Como você avalia a contribuição do(s) treinamento(s) para a sua prática clínica?

- ☐ Muito boa
- ☐ Boa
- ☐ Regular
- ☐ Ruim
- ☐ Muito ruim

28. Com que frequência você busca informações sobre TB?

- ☐ Nunca
- ☐ Quase nunca
- ☐ As vezes
- ☐ Quase sempre
- ☐ Sempre

AVALIAÇÃO DO AMBIENTE

29. A área de atendimento clínico que você utilza da Unidade possui ventilação adequada?(fluxo de ar)

☐ Sim ☐ Não

30. A recepção da Unidade possui ventilação adequada?(fluxo de ar)

☐ Sim ☐ Não

31. Número de janelas/similares que ficam abertas diariamente para ventilação do ambiente:

32. Número de portas/similares que ficam abertas diariamente para ventilação do ambiente:

FATORES DE RISCO

33. Você possui alguma comorbidade ou faz uso de alguma droga imunossupressora?

☐ Sim ☐ Não

33a. Se sim, qual?

17740

☐☐☐ - ☐☐☐☐
iniciais número do estudo

FATORES DE RISCO

34. Você alguma vez fumou?

☐ Sim

☐ Não (vá para a questão 35)

34a. Se sim, fuma agora?

☐ Sim ☐ Não

34b. Anos que fumou ☐☐

34c. Número médio de cigarros/dia ☐☐

34. Você alguma ingeriu bebida alcóolica?

☐ Sim

☐ Não (vá para a questão 36)

35a. Se sim, bebe agora?

☐ Sim ☐ Não

35b. Anos que bebeu ☐☐

35c. Se sim, frequência que bebe:

☐ Raramente (,1dia/semana)

☐ Ocasionalmente (1-3 dias/semana)

☐ Frequentemente (4 a 6 dias/semana)

☐ Diariamente

☐ Recusou responder

HISTÓRIA ANTERIOR RELACIONADA A TB

36. Você já realizou teste tuberculínico?

☐ Sim. Quando? ☐☐ / ☐☐ / ☐☐☐☐ (DD / MM / AAAA)

☐ Não

☐ Não sabe

36a. Resultado do teste tuberculínico: ☐☐ mm

37. Você já recebeu terapia preventiva de TB?

☐ Sim ☐ Não ☐ Não sabe

38. Você está em tratamento ou já tratou TB?

☐ Sim ☐ Não

INVESTIGAÇÃO

39. A cicatriz de BCG está presente?

☐ Sim ☐ Não ☐ Incerta

40.O resultado do Raio X é suspeito para TB?

☐ Sim ☐ Não ☐ Incerto

41. O teste HIV foi realizado?

☐ Sim ☐ Não ☐ Recusa

41a. Resultado do teste HIV:

☐ Negativo

☐ Positivo

☐ Indeterminado

42A coleta de sangue foi realizada?

☐ Sim ☐ Não ☐ Recusa

43. Comentários:

PARA USO DO COLETOR DE DADOS

Iniciais ☐☐☐

Data da coleta: ☐☐ / ☐☐ / ☐☐☐☐ (DD / MM / AAAA)

Assinatura: ________

PARA USO DO DIGITADOR

Iniciais ☐☐☐

Data do registro: ☐☐ / ☐☐ / ☐☐☐☐ (DD / MM / AAAA)
